# Application of Sustainable Natural Resources in Agriculture: Acaricidal and Enzyme Inhibitory Activities of Naphthoquinones and Their Analogs against *Psoroptes cuniculi*

**DOI:** 10.1038/s41598-018-19964-0

**Published:** 2018-01-25

**Authors:** Xiao-Fei Shang, Ying-Qian Liu, Xiao Guo, Xiao-Lou Miao, Cheng Chen, Jun-Xiang Zhang, Xiao-Shan Xu, Guan-Zhou Yang, Cheng-Jie Yang, Jun-Cai Li, Xiao-Shuai Zhang

**Affiliations:** 1 School of Pharmacy, Lanzhou University, 222 South Tianshui Road, Lanzhou, 730000 P. R. China; 2 Key Laboratory of New Animal Drug Project, Gansu Province, Key Laboratory of Veterinary Pharmaceutical Development of Ministry of Agriculture, Lanzhou Institute of Husbandry and Pharmaceutical Sciences, Chinese Academy of Agricultural Sciences, 335 Jiangouyan, Lanzhou, 730050 P. R. China; 3 Tibetan Medicine Research Center of Qinghai University, Qinghai University Tibetan Medical College, Qinghai University, 251 Ningda Road, Xining, 810016 P. R. China

## Abstract

As important secondary plant metabolites, naphthoquinones exhibit a wide range of biological activities. However, their potential as sustainable alternatives to synthetic acaricides has not been studied. This study for the first time investigates the acaricidal activity of naphthoquinones against *Psoroptes cuniculi in vitro*. Furthermore, the *in vivo* activity, the skin irritation effects, the cytotoxicity and the inhibitory activities against mite acetylcholinesterase (AChE) and glutathione S-transferase (GST) of the two compounds that displayed the best insecticidal activity *in vitro* were evaluated. Among fourteen naphthoquinones and their analogs, juglone and plumbagin were observed to possess the strongest acaricidal activities against *P. cuniculi* with LC_50_ values of 20.53 ppm and 17.96 ppm, respectively, at 24 h. After three treatments, these two chemicals completely cured naturally infested rabbits in vivo within 15 days, and no skin irritation was found in any of the treated rabbits. Compared to plumbagin, juglone presented no or weak cytotoxicity against HL-7702 cells. Moreover, these two chemicals significantly inhibited AChE and GST activity. These results indicate that juglone has promising toxicity against *P. cuniculi*, is safe for both humans and animals at certain doses, and could be used as a potential alternative bio-acaricide for controlling the development of psoroptic mange in agricultural applications.

## Introduction

Quinones, an important class of secondary plant metabolites that includes benzo-, naphtho-, phenanthra-, and anthraquinones, are common in nature and have extensive pharmacological activities, such as antitumor, antiparasitic, antibacterial, insecticidal, fungicidal, anti-inflammatory, antipyretic, antiproliferative and cytotoxic effects^1–4^. Among these compounds, naphthoquinones, a group of highly reactive phenolic compounds, are extremely useful for the development of potent agrichemicals because of their significant antiparasitic and insecticidal activities^5–7^. This is especially true of 1,4-naphthoquinone and two of its derivatives, juglone and plumbagin, which have garnered considerable research and applied interests due to their broad range of potential biological activities; extracts derived from walnut husk residues showed strong nematicidal activity against the root-knot nematode *Meloidogyne hispanica*^8^.

Acariasis is a parasitic infection of the body surface or epidermis of animals that can reduce the productivity and quality of afflicted animals and is often fatal^9,10^. As a common ectozoic parasite infection in rabbits, *Psoroptes cuniculi* infestation causes intense pruritus and the formation of crusts and scabs in the external ear canal and the internal surfaces of the pinna^11^. Rabbit psoroptic mange has become a global disease that causes considerable losses in the United States, Italy, Turkey, South Korea, India and China^12–17^. To treat and control psoroptic mange, synthetic acaricides have been widely applied and have exhibited relatively satisfactory treatment efficacy. However, acaricide resistance, drug residues, environmental pollution, health disorders and other side effects have been inadvertently caused by certain acaricides currently in use^18^, and acaricide residues further threaten the safety of animal-derived food. Hence, to address these issues and stimulate the development of safer and sustainable alternative drugs, considerable effort has been made to exploit natural acaricides from plants, animals and microorganisms.

Many plants, including *Azadirachta indica, Adonis coerulea*, *Eugenia caryophyllata*, and *Eupatorium adenophorum*, have a high toxicity to *P. cuniculi* and have shown significant clinical efficacy when used in the clinic to control animal acariasis^19–22^; the advantages of this approach include high efficiency, lower resistance, and decreased pollution. Furthermore, several active compounds have been isolated, such as eugenol, δ-cadinene, carvacrol, octadecanoic acid-tetrahydrofuran- 3,4-diylester, and 9-oxo-10,11-dehydroageraphorone, among others, which were used as lead compounds to develop potential acaricides^13,23–26^. However, the acaricidal activities of naphthoquinones against *P. cuniculi* have not been reported, and their possible mechanisms of action are currently unknown.

**Figure 1:**
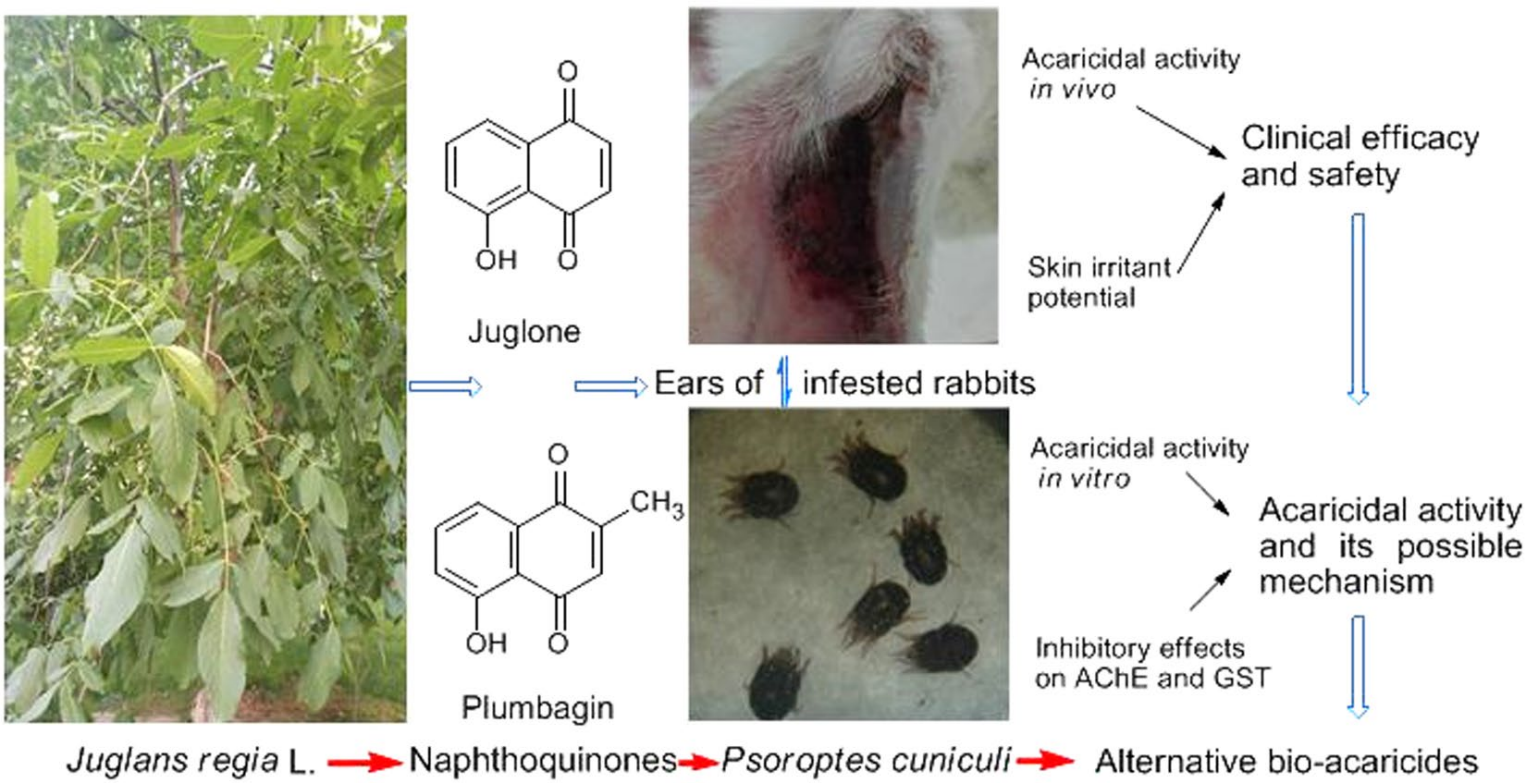
Juglone has promising toxicity against *Psoroptes cuniculi* and could be used as a potential bio-acaricide.

**Figure 2:**
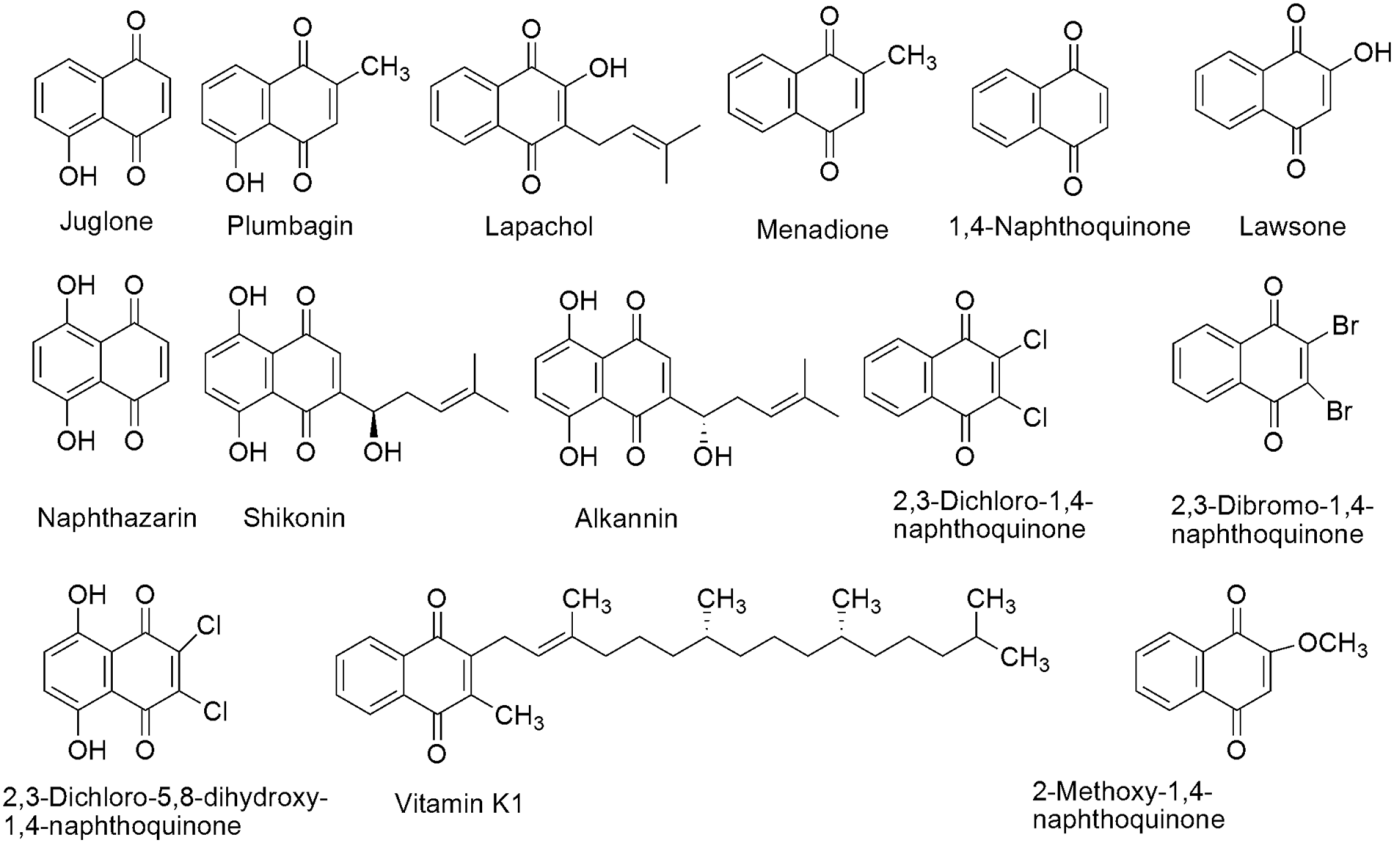
Chemical structures of the naphthoquinones used in the test.

In this paper, we aimed to study the acaricidal activity of fourteen naphthoquinones and their analogs (Figs 1,2) against *P. cuniculi in vivo* and explore their structure-activity relationships. Then, the two naphthoquinones with the best toxicity *in vivo* were evaluated for clinical use by determining their *in vitro* acaricidal activity, skin irritation effects and cytotoxicity against HL-7702 cells. The mechanism of actions of these two chemicals were investigated by elucidating their inhibitory activities against acetylcholinesterase (AChE) and glutathione S-transferase (GST) to guide the development of new plant-based acaricides.

## Results

### Acaricidal activity of naphthoquinones *in vitro*

Among the fourteen naphthoquinones and their analogs, juglone and plumbagin presented the strongest acaricidal activities against *P. cuniculi* with LC_50_ values of 20.53 ppm and 17.96 ppm, respectively, at 24 h. At 100 ppm, two chemicals killed all the tested mites (100% mortality) at 24 h. The next best compounds in terms of efficacy were 2,3-dichloro-5,8-dihydroxy-1,4-naphthoquinone, naphthazarin, 2,3-dichloro-1,4-naphthoquinone, 1,4-naphthoquinone, 2,3-dibromo-1,4-naphthoquinone and lapachol, all of which had LC_50_ values less than 100 ppm. Shikonin, lawsone, alkannin and vitamin K1 exhibited weak acaricidal activities with LC_50_ values greater than 1000 ppm at 24 h. The LC_50_ value for the positive control ivermectin against mites was 3.38 ppm (Table 1).

**Table 1:** The LC_50_ values of naphthoquinones against *Psoroptes cuniculi in vitro* by CLL model.

No.	Compounds	Regression line	LC_50_* (ppm)	95% CI** (ppm)	Pearson Chi-square
1	Juglone	Y = 5.045X-6.620	20.53	17.94–23.34	2.802
2	Plumbagin	Y = 4.634X-5.812	17.96	15.48–20.57	2.002
3	Menadione	Y = 3.626X-9.305	368.27	255.79–687.78	9.752
4	1,4-Naphthoquinone	Y = 2.034X-3.3719	67.35	24.17–217.38	29.625
5	Naphthazarin	Y = 2.765X-4.643	47.79	21.64–112.37	25.247
6	Shikonin	—	>1000	—	—
7	Alkannin	—	>1000	—	—
8	Lapachol	Y = 2.632X-5.040	82.15	69.14–97.91	4.440
9	Vitamin K1	—	>1000	—	—
10	Lawsone	—	>1000	—	—
11	2,3-Dichloro-5,8-dihydroxy-1,4-naphthoquinone	Y = 3.091X-4.492	28.42	24.00–33.35	3.022
12	2-Methoxy-1,4-naphthoquinone	Y = 3.445X-7.697	169.53	125.58–233.61	7.087
13	2,3-Dichloro-1,4-naphthoquinone	Y = 2.093X-3.545	49.38	27.45–85.77	12.825
14	2,3-Dibromo-1,4-naphthoquinone	Y = 1.900X-3.622	80.60	41.73–168.27	15.875
15	Ivermectin	Y = 1.616X-0.855	3.38	2.59–4.31	6.381

^*^LC_50_ was analyzed according to the mortality (%) of mites at 24 h.

^**^CI, confidential interval.

### Acaricidal activity of juglone and plumbagin *in vivo*

**Figure 3:**
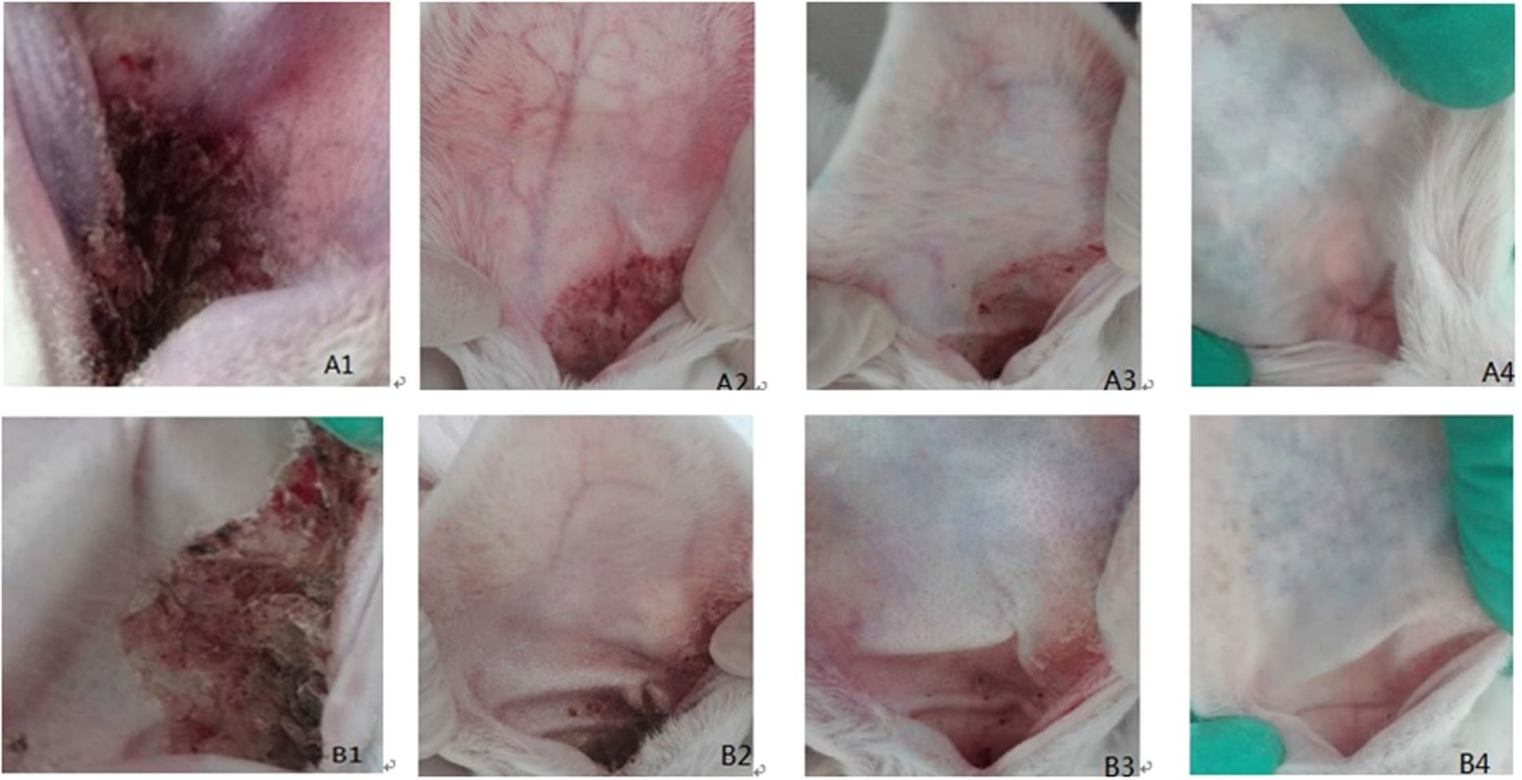
Clinical observations of the rabbits treated with 50 ppm juglone (A1–A4) and plumbagin (B1–B4) at 0, 5, 10, 15 days.

Before treatment, no difference was observed between the three selected groups, with most of the rabbits exhibiting mites and half of their pinna filled with scabs (*P* > *0.05*). After the treatments, the scabs in the rabbit ears treated with the two compounds were substantially decreased, with significant differences between the treatment groups and the control group at day 5 (*P* < *0.01*). In addition, at day 10, only small scabs or secretions existed in the ear canals of the juglone and plumbagin treatment groups with clinical scores of 0.8 and 0.7, respectively. Finally, at day 15, the rabbits were free of mites and showed positive mental and physical status with normal movement ability, good appetite for food and good fur and skin (clinical scores of 0.2 and 0.1 for the juglone and plumbagin groups, respectively). However, the status of the rabbits in the untreated control group declined, and they became emaciated, requiring clinical treatment at day 10 (Table 2; Fig. 3). These results indicated that the two compounds presented marked acaricidal activity *in vivo* and that they could be used in the clinic to treat and control psoroptic mange.

**Table 2:** Acaricidal activity of juglone and plumbagin against *Psoroptes cuniculi* infestation in rabbits, measured by clinical score.

Groups	Day(s)			
	0	5	10	15
Juglone (50 ppm)	4.0 ± 0.71	2.4 ± 0.55**	0.8 ± 0.27**	0.2 ± 0.27
Plumbagin (50 ppm)	3.8 ± 0.84	2.4 ± 0.55**	0.7 ± 0.27**	0.1 ± 0.22
Control	3.8 ± 0.45	3.8 ± 0.45	4.0 ± 0.00	—^#^

Each value represents the mean ± S.E.M. of 5 rabbits.

^**^P < *0.01* compared with the control.

^#^After the observation at day 10, rabbits in the control group were treated.

### Skin irritant potential and cytotoxicity

As shown in Table 3, during the experimental periods in a skin irritant potential test, no skin irritation, including inflammatory responses, irritation, and allergy was found in the healthy rabbits or in the naturally infested rabbits treated with juglone and plumbagin and the control group, such as inflammatory response, irritation, and allergy, and the scores of skin irritation intensity were zero in all groups (Table 3). In a cytotoxicity test, compared to juglone (IC_50_ > 100 µg/ml), plumbagin presented moderate toxicity against HL-7702 normal liver cells (IC_50_ of 4 µg/ml). This result indicated that the latter compound may affect the activity of liver cells when it is absorbed and transported to the human liver (Table 3).

**Table 3:** The skin irritant potential and cytotoxicity against HL-7702 cells of juglone and plumbagin.

Group	Skin irritant potential (15 days)*			IC_50_ (µg/ml)
	Healthy rabbits	Naturally infested rabbit	Control group	HL-7702 cell
Juglone	No irritation	No irritation	No irritation	>100
Plumbagin	No irritation	No irritation	No irritation	4

^*^The grading standard for skin irritation or allergic response of ref.^57^ was adopted.

### Inhibition of acetylcholinesterase activity

**Figure 4:**
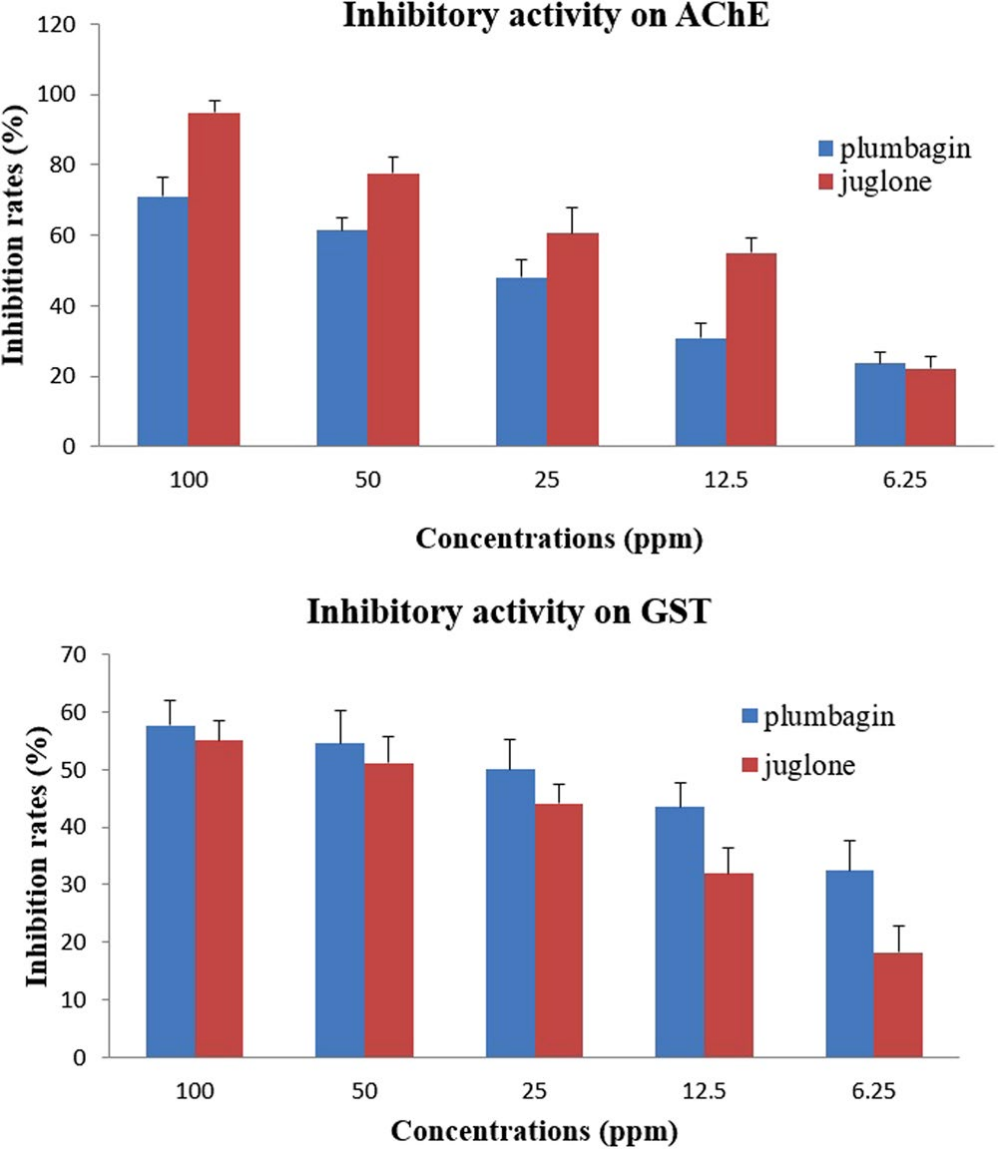
Inhibitory effects of plumbagin and juglone against mite AChE and GST.

Figure 4 shows that both chemicals exhibited significant AChE inhibitory activities with inhibition ratios of 70.99%, 61.37%, 47.98%, 30.74% and 23.56% for plumbagin and 94.85%, 77.62%, 60.54%, 54. 97% and 22.20% for juglone at concentrations of 100 ppm, 50 ppm, 25 ppm, 12.5 ppm and 6.25 ppm, respectively. The corresponding IC_50_ values based on the inhibition ratios of plumbagin and juglone were 29.87 ppm and 14.81 ppm, respectively (Table 4). Juglone presented better AChE inhibitory activity than plumbagin, which may be related to the acaricidal activity of the two chemicals.

**Table 4:** IC_50_ values of the AChE and GST inhibitory activity of plumbagin and juglone.

Chemicals	Enzymes	IC_50_ (ppm)	95% CL* (ppm)	Regression line	Pearson Chi- square
Juglone	AChE	14.81	7.76–22.89	Y = 1.715X-2.008	7.845
	GST	52.49	37.58–86.23	Y = 0.830X-1.427	2.790
Plumbagin	AChE	29.87	23.55–38.64	Y = 1.113X-1.642	0.688
	GST	32.69	19.70–64.52	Y = 0.521X-0.789	1.026

^*^Confidence limit.

### Inhibition of glutathione S-transferase activity

As shown in Fig. 4, juglone and plumbagin exhibited strong GST inhibitory activities with inhibition ratios of 57.68%, 54.63%, 49.99%, 43.46% and 32.50% for plumbagin and 55.00%, 51.16%, 44.16%, 31.98% and 18.30% for juglone at concentrations of 100 ppm, 50 ppm, 25 ppm, 12.5 ppm and 6.25 ppm, respectively. The corresponding IC_50_ values based on the inhibition ratios of plumbagin and juglone were 32.69 ppm and 52.49 ppm, respectively (Table 4).

## Discussion

Naphthoquinones are an important class of secondary plant metabolites that are widespread in nature and that have been shown to possess antitumor, antimicrobial, antifungal, antifeedant, insecticidal and acaricidal activities^27–33^. However, the biological mechanisms of the effects of naphthoquinones and their structure-activity relationships are still not explained completely. In this paper, we investigated the acaricidal activities of naphthoquinones against *P. cuniculi in vivo* and the *in vitro* activity of two of these chemicals, and our results may provide insights into identifying potential naphthoquinone-based acaricides.

Among the fourteen naphthoquinones and their analogs tested, juglone and plumbagin possessed the strongest acaricidal activities against *P. cuniculi* with LC_50_ values less than 25 ppm *in vitro*. These compounds presented better acaricidal activity than most plant-based acaricides^13,23–26^. *In vivo*, juglone and plumbagin at 50 ppm presented similar therapeutic effects when administered to rabbits infested with *P. cuniculi*. Structure-activity relationship analysis revealed that a hydroxyl group at the 5- or 8- position is necessary for acaricidal activity, as chemicals containing one or both of these hydroxyls had robust activity. This relationship also holds for the insecticidal, antimalarial and anticancer activities of napthoquinones^34–37^, where the presence of the 5-hydroxyl group is crucial for inhibition. Meanwhile, plumbagin is characterized by the presence of a 5-hydroxyl group and a 2-methyl group, whereas juglone lacks the latter group. These differences are sufficient to lead to distinct single/two-electron reduction potentials in aqueous media. Thus, the activity of juglone is better than plumbagin, which is related to their ability to promote redox cycling/oxidative stress. Moreover, the acaricidal activity is dramatically reduced when juglone is substituted with several common functional groups^38^, and 2,3-dichloro-1,4-naphthoquinone and 2,3-dibromo-1,4-naphthoquinone were significantly less active than juglone. Aliphatic side chains in the 3-position and a substituent in the 2-position increase the chemical stability by preventing the oxidation of the carbonyl oxygen and hydroxyl groups, but when the length of carbon chain exceeds six atoms, the activity is decreased^28^, as observed for vitamin K1, shikonin and alkannin. Because 2-hydroxy-1,4-naphthoquinone had lowest Log P value among the naphthoquinones due to presence of the hydroxyl group^39^, the LC_50_ value of lawsone was greater than 1000 ppm.

Pesticides have several insecticidal mechanisms but primarily kill by interfering with enzyme activity related to insect metabolism^19,25^. AChE, related to the vital movements of insects, plays an important role in insect neural conduction^40^, and the inhibition of AChE is one of the modes of action of many insecticides, such as organophosphates and carbamates. A variety of compounds derived from plants and fungi are also known to inhibit AChE activity^41^. In addition, GST plays a central role in the detoxification of xenobiotic and endogenous compounds in mites^42,43^ and is partly responsible for the development of resistance to certain chemicals^44,45^. Our results indicated that juglone demonstrated stronger AChE inhibitory activity than plumbagin and that the acaricidal activities of juglone and plumbagin are likely related to their AChE inhibitory activity (IC_50_ values of 14.81 ppm and 29.87 ppm, respectively); thus, AChE may be the relevant target site of the two chemicals against mites^46^. Moreover, the inhibitory activity against GST would be predicted to further induce mite death, and the different IC_50_ values likely stem from the different substituents at the 2-position in these quinones. Due to their different mechanisms of action, the GST inhibitory activity of juglone is weaker than that of plumbagin. Plumbagin stoichiometrically converts GSH into GSSG, whereas for juglone, much of the GSH lost does not appear as GSSG, suggesting that the decrease in cellular glutathione is due to the arylation mechanism^47^.

In the skin irritant potential test, no skin irritation was found in any of the tested healthy rabbits or naturally infested rabbits treated with juglone and plumbagin (50 ppm) during the experimental periods. Although the two chemicals did not show any skin irritant potential in the above test, the toxicity of juglone and plumbagin against human or animal tissue when they were absorbed by skin remain unclear. Many previous studies have reported the cytotoxicity of juglone and plumbagin against some cell lines^8,48–51^, but most of the cells were evaluated in *in vitro* and were cancerous cells. To evaluate their safety for clinic use, the cytotoxicity of the two chemicals against HL-7702 normal liver cells was studied. Juglone presented no or weak toxicity against liver cells, but plumbagin exhibited moderate toxicity with an IC_50_ values of 4 µg/ml. Overall, these results indicated that at the given doses, juglone and plumbagin are safe for both human and animal skin, whereas plumbagin may have potentially adverse effects on liver tissue in both humans and animals. Thus, the toxicity of plumbagin should be studied further, and how to apply it properly in the clinic should also be investigated.

In conclusion, juglone exerts promising toxicity against *P. cuniculi in vitro* and appears relatively safe for both skin and liver cells at certain doses. It can be used to treat naturally infested rabbits *in vivo*. This study lays the foundation for future development of juglone as a natural acaricide to control psoroptic mange in agricultural applications.

## Experimental Section

### Mites

*Psoroptes cuniculi* was isolated from the ear cerumen and scabs and toes of naturally infested rabbits. Ear cerumen and scabs were collected and placed in Petri dishes and then incubated at 35 °C for 30 min in an incubator. *P. cuniculi* was then collected and examined using a light microscope and was identified according to morphological features^52–55^. After collecting the materials, the rabbits were treated immediately. The experiments complied with the rulings of Gansu Experimental Animal Center (Gansu, China) and were approved by the Ministry of Health, P.R. China, in accordance with NIH guidelines.

### Chemicals

Naphthoquinones and their analogs (Fig. 2) were purchased from the following companies: juglone (97%), lapachol (97%), 2,3- dichloro-1,4-naphthoquinone (98%), vitamin K1 (98%), and naphthazarin (98%) were purchased from Aladdin Industrial Co. Ltd. (Shanghai, China); plumbagin (97%) was purchased from J&K Scientific Ltd. (Beijing, China); menadione (98%), 1,4-naphthoquinone (99%), shikonin (97%) and lawsone (98%) were purchased from Meryer Chemical Technology Co. Ltd. (Shanghai, China); 2,3-dichloro-5,8-dihydroxy-1,4-naphthoquinone (97%) was purchased from Tokyo Chemical Industry Co. Ltd. (Tokyo, Japan); 2-methoxy-1,4-naphthoquinone (98%), 2,3-dibromo-1,4-naphthoquinone (97%), acetylthiocholine iodine, 5,5′-dithiobis(2-nitrobenzoic acid), L-reduced glutathione and 1-chloro-2,4-dinitrobenzene were purchased from Sigma-Aldrich Co. Ltd. (St. Louis, USA) and from Toronto Research Chemical Inc. (Toronto, Canada); and alkannin (98%) and ivermectin (94%) were purchased from Shanghai Yuanye Biotechnology. Co. Ltd. (Shanghai, China).

### Acaricidal activity of naphthoquinones *in vitro*

*In vitro* experiments were carried out according to previously described methods^20,23^. At concentrations of 500 ppm, 250 ppm, 100 ppm, 50 ppm, 25 ppm, and 10 ppm, 300 µl solutions of the fourteen naphthoquinones were separately added into culture plates (diameter of 60 mm), and filter papers were used to absorb the excess liquid. As a positive control, ivermectin was applied at a concentration range from 50 to 1 ppm. All chemicals were diluted in 10% DMSO, and 10% DMSO was also added to the untreated group. Next, 10 adult *P. cuniculi* mites were collected with a needle from the ear cerumen of naturally infested rabbits and placed in each well. All plates were incubated at 25 ± 1 °C under 75% relative humidity in an incubator (Chongqing YESI Co. Ltd., China). After 24 h of treatment, mortality was assessed. The viability of the mites was checked regularly via stimulation with a needle, and the mites were recorded as dead if the body and appendages did not move under a microscope. All treatments were replicated five times.

### Acaricidal activity of juglone and plumbagin *in vivo*

*In vivo* experimental procedures from previously described methods^56^ were modified and performed. Fifteen naturally infested New Zealand rabbits with similar ages and weights were examined and randomly divided into three groups; there were no significant differences in the clinical scores in the ears between the groups of the rabbits after clinically evaluating the extent of the scabbing in the external ear canal. Prior to treatment, none of the rabbits had been treated with any acaricides or other agents, and no other complicating diseases were observed in the rabbits. The scoring system used to evaluate the degree of infestation is presented in Table 5^56^.

**Table 5:** The scoring system used to determine the clinical scores of infection and the degree of recovery.

Degree of infection and recovery	Clinical score
Absence of scabs and mites	0
Irritation in the ear canal but no mites observed	0.5
Small number of scabs in the ear canal, mites present	1
External ear canal filled with scabs, mites present	2
Scabs in ear canal and proximal 1/4 of pinna, mites present	3
1/2 of the pinna filled with scabs, mites present	4
3/4 of the pinna filled with scabs, mites present	5
All of the internal surface of the pinna filled with scabs	6

^*^The table was adopted from ref.^56^.

For this assay, all the treatments were topical. A concentration of 50 ppm of juglone and plumbagin was selected to evaluate the acaricidal activity, and 2 ml solutions were sprayed directly into the rabbits’ pinna and in the external ear canal, especially in the areas with cerumen and/or scabs. All chemicals were diluted in 10% DMSO, and the animals in the control group were treated with 2 ml of 10% DMSO. These drugs were applied three times at 0, 5 and 10 days. Subsequently, on days 0, 5, 10 and 15 after the beginning of treatment, all rabbits’ ears were examined with an otoscope in the clinic to evaluate the presence of scabs, and the cerumen and/or scabs were collected from each ear of each rabbit using dissecting forceps to evaluate the presence of mites under a light microscope. During the experimental periods, the presence of clinical signs was assessed daily, and any abnormal reactions were recorded.

### Skin irritant potential

Experimental procedures were performed as previously described^57^. Fifteen healthy rabbits of similar ages and weights were divided into three groups. The control group was treated with 2 ml of 10% DMSO, and the other groups were treated topically with 50 ppm of juglone and plumbagin, which were diluted in 10% DMSO in the rabbit’s ear. As described for the method of the acaricidal test *in vivo*, these drugs were applied three times at 0, 5 and 10 days. Subsequently, on days 0, 5, 10 and 15 after the beginning of treatment, the skins of all the rabbits’ ears were examined, and skin reactions were recorded. Meanwhile, the skin reactions of all naturally infested rabbit’s ears in the acaricidal test *in vivo* were also recorded.

### MTT test

Experiments were carried out according to previously described methods^30,31^. The normal human liver cell line HL-7702 was purchased from the Procell life Sci & Tech. Co. Ltd. (Wuhan, China). Cells were grown in supplemented 1640 medium, and the cell cultures were maintained at 37 °C in a humidified CO_2_ (5%) incubator. Cells were cultured at a density of 5000 cells/well into a 96-well plate and treated with juglone and plumbagin at various concentrations for 48 h. Then, a fresh solution of MTT (0.5 mg/ml) was added to each well and incubated for an additional 4 h. After that, the supernatants were discarded, followed by the addition of 150 μl of DMSO and vibration for 10 min. The absorbance was measured at 570 nm using a BIO-RAD model 680 multi-well plate reader. Three replicates were performed for each group.

### Inhibition of acetylcholinesterase and glutathione S-transferase

Experiments were carried out according to a previously described method^58–60^. First, 200 mites (*P. cuniculi*) were collected, immediately placed in a homogenizer and homogenized for 5 min with 600 μl of phosphate buffer in ice water. Then, the homogenates of the mites were transferred to centrifuge tubes and centrifuged at 10000 × g at 4 °C for 10 min, and the supernatants were carefully collected into tubes and used as the enzyme source.

Juglone and plumbagin were prepared in 10% DMSO solution at concentrations of 100 ppm, 50 ppm, 25 ppm, 12.5 ppm and 6.25 ppm, and 100 µl of these chemicals were added to a 96-well microplate with 100 µl of crude protein. A solution of 10% DMSO without either of the two chemicals was used as a control.

For AChE activity, after a preincubation period of 10 min at 37 °C, 10 µl of 10 mM acetylthiocholine iodine (ASChI) and 10 µl of 4 mM 5,5′-dithiobis (2-nitrobenzoic acid) (DTNB) were added to a 96-well microplate and were diluted with phosphate buffer. Then, the AChE inhibitory activity was assayed by determining the maximum velocity (*V*_*ma*x_) for 30 min at 40 s intervals at 405 nm at room temperature with a microplate reader (ELISA) (Multiskan MK3, Thermo Scientific, U.S.A.). These tests were performed in triplicate.

For GST activity, after a preincubation period of 10 min at 37 °C, 100 µl of substrate solution (4 ml of 10 mM reduced glutathione in phosphate buffer with 1 ml of 10 mM 1-chloro-2,4-dinitrobenzene (CDNB) in methanol) was added to a 96-well microplate. Then, the GST inhibitory activity was assayed by determining the *V*_*ma*x_ for 30 min at 40 s intervals at 340 nm at room temperature with a microplate reader.

For both enzymes, inhibitory activity (%) was calculated as 100 − (*V*_*ma*x_ of treatment/*V*_*ma*x_ of control × 100).

### Statistical analysis

Data were analyzed using SPSS software version 18.0 and expressed as the means ± SD. Data were analyzed using one-way ANOVA, followed by Student’s two-tailed t-test for comparisons between the test and control groups and Tukey’s test when the data involved three or more groups. P-values of less than 0.05 (P < 0.05) were considered significant. The median lethal concentration value (LC_50_) was calculated by probit analysis.
